# Effect of Cross-Linking on the Performances of Starch-Based Biopolymer as Gel Electrolyte for Dye-Sensitized Solar Cell Applications

**DOI:** 10.3390/polym9120667

**Published:** 2017-12-01

**Authors:** Pavithra Nagaraj, Asija Sasidharan, Velayutham David, Anandan Sambandam

**Affiliations:** 1 Nanomaterials and Solar Energy Conversion Lab, Department of Chemistry, National Institute of Technology, Tiruchirappalli 620 015, India; pavithra1516@gmail.com (P.N.); asijasasidharan@gmail.com (A.S.); 2 Electro Organic Division, CSIR-Central Electrochemical Research Institute, Karaikudi 630 006, India; dvelayutham@cecri.res.in

**Keywords:** starch-based biopolymer, crosslinking, morphology, conductivity, dye-sensitized solar cells

## Abstract

Dye-sensitized solar cells (DSSCs) have become a validated and economically credible competitor to the traditional solid-state junction photovoltaic devices. DSSCs based on biopolymer gel electrolyte systems offer the perspective of competitive conversion efficiencies with a very low-cost fabrication. In this paper, a new starch-based biopolymer gel electrolyte system is prepared by mixing lithium iodide and iodine with bare and citric acid cross-linked potato starches with glycerol as the plasticizing agent. The effect of the preparation methods on the starch cross-linking degree as well as the photoconversion efficiency of the resulting DSSC cells is carefully analyzed. Fourier transform spectroscopy, X-ray diffraction, and scanning electron microscopy were used to characterize the morphology and conformational changes of starch in the electrolytes. The conductivity of the biopolymer electrolytes was determined by electrochemical impedance spectroscopy. DSSC based on the starch-gel polymer electrolytes were characterized by photovoltaic measurements and electrochemical impedance spectroscopy. Results clearly show that the cross-linking increases the recombination resistance and open circuit voltage (*V*_OC_) of the DSSC, and thereby the photoconversion efficiency of the cell. In particular, electrolytes containing 1.4 g bare and cross-linked starches showed ionic conductivities of *σ* = 1.61, 0.59, 0.38, and 0.35 S cm^−1^, and the corresponding DSSCs showed efficiencies of 1.2, 1.4, 0.93, and 1.11%, respectively.

## 1. Introduction

The energy crisis is the most important global issue that human society is currently facing. This problem is mainly related to three factors: increasing energy demand to support the economy, depletion of fossil fuels, and the greenhouse effect due to combustion of fossil fuels [1,2]. There are many renewable energy resources available in nature, such as the tide, the wind, the sun, and geothermal energy [3]. Among these, solar energy is a very reliable source for achieving energy demands because it is environmentally-friendly, durable, and enormous. The energy of the sun is converted to different forms of energy by several techniques, including photovoltaics, solar thermal techniques, etc., among which photovoltaics is a powerful tool for solar energy conversion [4]. Solar cells are one of the promising devices for converting solar energy directly into electrical energy. Among various types of solar cells, dye-sensitized solar cells (DSSCs), which adopt the principle of artificial photosynthesis introduced by O’Regan and Gratzel in the 1990s, is an low-cost alternative to traditional photovoltaic devices [5]. The property of separating the functions of light absorption and charge transfer makes DSSCs more efficient than conventional semiconductor devices [5].

Basically, a typical DSSC consists of five components: a substrate (i.e., a glass plate coated with fluorine-doped tin oxide, FTO), a semiconductor monolayer such as TiO_2_, ZnO, or SnO_2_, and a dye sensitizer together which constitute the photoanode, an electrolyte containing a redox couple, and a counter-electrode. The mechanism of DSSCs involves the photo-excitation of the sensitizer and the injection of a photo-excited electron into the conduction band of the semiconductor oxide, generating the oxidized form of the dye. The electron from the conduction band of the semiconductor oxide layer reaches the substrate and flows through the external circuit. Then, the ground state of the dye is regained by electron donation from the reduced state of the electrolyte, which in turn is regenerated by the reduction of the oxidized species of the electrolyte at the counter-electrode. DSSCs can be produced at reasonably low cost while yielding relatively decent efficiencies [6]. Researchers are focusing on how to improve the system by changing the components (i.e., dyes, electrolytes, anode materials, etc.) [7].The electrolyte is one of the important components of DSSCs.

Liquid electrolytes (LEs) prepared by dissolving a redox couple in low-boiling organic solvents are conventionally used in DSSC because of easy preparation, high conductivity, low viscosity, and good interfacial wetting between electrolytes and electrodes, and thus attaining high conversion efficiency. Liquid electrolytes have limitations including packing difficulties, leakage, corrosion of electrodes, etc. Over time, polymer electrolytes (PEs) have entered the picture to overcome the difficulties faced by liquid electrolytes. Solid polymer electrolyte (SPE) allows the movement of ions without the need for a liquid or soft membrane separating the electrodes. Due to the hard matrix, solid electrolytes have low conductivity, and conversion efficiencies are very low when compared with liquid electrolytes. Gel polymer electrolytes (GPEs) possess both the cohesive properties of a solid and the diffusive properties of a liquid, and they have a low vapour pressure, which assures long-term stability and better efficiency.

Though PEs, with many synthetic polymer electrolytes, are reported with high efficiency, research aiming to find new biopolymer electrolytes is ongoing due to the environmental effects caused by synthetic polymers. Natural polymers like chitin, cellulose, gelatin, and chitosan are being successfully evaluated for this purpose. Cellulose grafted homogeneously with acrylic acid by in situ polymerization in an ionic liquid (1-butyl-3-methylimidazolium iodide) as reaction medium has shown an efficiency of 5.51% [8]. Biopolymer composite membranes based on chitosan doped with the ionic liquid (IL) 1-ethyl 3-methyl-imidazolium-thiocyanate (EMImSCN) obtained an efficiency of 0.73% [9]. A biodegradable rice starch-based solid polymer electrolyte incorporated with 20 wt % 1-methyl-3-propylimidazolium iodide (MPII) ionic liquid and sodium iodide salt prepared via solution cast technique showed an efficiency of 2.09% [10]. However, starch-based gel polymer electrolyte has yet to be reported.

**Figure 1:**
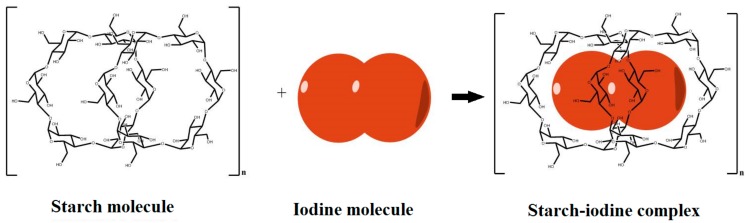
Schematic representation showing the formation of the starch-iodine complex.

Starch an easily available biopolymer. However, the problem in using a starch-based biopolymer electrolyte in DSSCs is the possibility of the formation of a starch–iodine complex, because the commonly used redox couple in DSSC is iodide/triiodide. When starch comes in contact with I_2_, in the presence of atmospheric moisture, iodine molecules will be inserted into the cage-like structure of amylopectin and form an array of amylose chains, forming a starch-iodine complex (Figure 1), which leads to the unavailability of free iodine in the electrolyte and significantly affects the conductivity of the electrolyte. Hence, a cross-linking approach is adopted to prevent the formation of the starch–iodine complex. Cross-linking of starch restricts the insertion of iodine molecules into the starch polymer chain, thus making iodine available for ionic transport. Three different procedures are adopted for cross-linking. Citric acid was chosen as the additive because of its multi-carboxylic structure, allowing it to interact with the hydroxyl groups of the starch.

In this work, a new biopolymer electrolyte system is prepared by mixing an iodide/iodine redox couple with bare and cross-linked (using citric acid) potato starches in the presence of glycerol as the plasticizing agent. The conformational changes of cross-linked starch as electrolytes were characterized by Fourier transform infrared spectroscopy (FT-IR). The effect of cross-linking on the crystallinity of starch was studied by X-ray diffraction spectroscopy (XRD) and differential scanning calorimetry (DSC). The conductivity of the cross-linked polymer gel electrolytes was determined by electrochemical impedance spectroscopy (EIS), and finally, photovoltaic measurements were done for DSSCs fabricated using the prepared electrolytes.

## 2. Materials and Methods

### 2.1. Materials

Dimethylformamide, glycerol, lithium iodide, starch, citric acid, Na_2_CO_3_, NaOH, poly(ethylene glycol) (PEG, *M*_w_ = 10,000), acetylacetone, TiCl_4_, and N719 Dye were purchased from Sigma-Aldrich, Bangalore, India and TiO_2_ (P25) was purchased from Degussa, Mie, Japan. All the reagents were purchased and used without any purification.

### 2.2. TiO_2_ Paste Preparation

TiO_2_ paste was prepared as per the procedure cited elsewhere [11]: 0.75 g of TiO_2_ (P25) nanoparticles and 0.024 g of PEG were added to a solution of 1 M HNO_3_. Then, 0.315 mL of acetylacetone and 0.06 mL of Triton-X was added to the mixture. Later, the mixture was ultrasonicated for 1 h, then stirred for 24 h until a homogeneous TiO_2_ paste was obtained. 

### 2.3. Preparation of Nanocrystalline TiO_2_ Films (Photoanodes) and Platinum Counter Electrodes

FTO plates were used as the substrate for photoanode and cathode. FTO glass plates were washed by sonicating with soap water, distilled water, acetone, and 2-propanol subsequently for 15 min. For photoanodes, washed FTO plates were pre-treated by dipping in a 40 mM solution of TiCl_4_ for 30 min at 70 °C. After TiCl_4_ treatment, the plates were washed with water, ethanol, and heated at 100 °C on a hot plate for 10 min. TiO_2_ paste was then coated on the substrate using doctor blade method. The nanocrystalline films were obtained by sintering at 450 °C for 30 min in a tubular furnace. The obtained films were sensitized by dipping in a N719 dye solution (3 × 10^4^ M) prepared in ethanol solvent for 24 h. Finally, the films were treated with a LiI solution in acetonitrile of low concentration [12]. This TiO_2_-coated FTO glass with an active area of 0.25 cm^2^ acted as the photoanode for the DSSCs. 

The Platinum (Pt)-coated FTO plate was prepared by drop casting 5 × 10^−4^ M solution of H_2_[Pt(Cl_6_)] in 2-propanol and sintering at 450 °C for 30 min in a tubular furnace, and this electrode was used as the counter electrode for the DSSC.

### 2.4. Preparation of Cross-Linked Starch

Starch was cross-linked by the following three different procedures, identified as A1, A2, and A3.

A1: 5 g of starch was mixed with 100 mL of distilled water, 2 mL glycerol, and 0.5 g of citric acid at room temperature for 5 min. This suspension was transferred to a water bath at 90 °C for 30 min and agitated by a magnetic stirrer. After cooling, the solution was poured into a Petri dish and dried at 50 °C. After drying, the product was weighed and used for electrolyte preparation [13].

A2: 0.5 g citric acid and 0.033 g Na_2_CO_3_ were mixed with 100 mL distilled water and stirred for 5 min. Five grams of starch was added to this solution and stirred on a hot plate at 90 °C. After 20 min, 2 mL of glycerol was added and stirred. The resulted homogeneous solution was poured into a Petri dish and dried. The dried product was used for the preparation of electrolyte [14]. 

A3: 0.5 g citric acid and 10 g of NaOH were dissolved separately in 25 mL water. Then, NaOH solution was added to the citric acid solution until the pH became 3.5, and 5 g starch was added to the solution and kept at room temperature for 12 h. This solution was stirred for 1 h and poured into a petri dish and kept for drying at 55 °C in a hot air oven [15].

In the case of A2 and A3, Na_2_CO_3_ and NaOH were added for effective cross-linking of starch with citric acid.

### 2.5. Preparation of Starch-Based Polymer Gel Electrolytes

**Figure 2:**
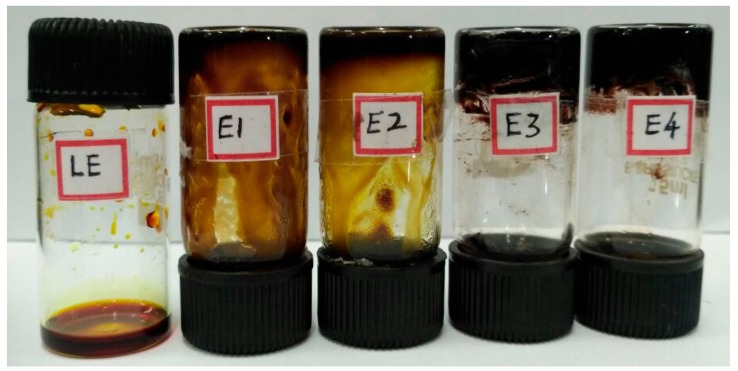
Prepared liquid electrolyte (LE) and electrolytes containing 1, 1.2, 1.4, and 1.6 starch (E1, E2, E3, and E4 respectively).

A homogeneous liquid electrolyte was prepared by thoroughly stirring 1 mL Dimethylformamide (DMF), 1 mL glycerol, 0.134 g LiI, and 0.0235 g of I_2_ in a glass vial for 1 h. The gelation of this liquid electrolyte was carried out by adding from 0 to 2 g starch in increments of 0.2 g. After adding starch, the system was kept under constant stirring for 2 h at 90 °C until a homogeneous mixture was obtained. The addition of starch to the liquid electrolyte from 0.2 to 1.2 g gave electrolytes with partially liquid character. The addition of 1.4 g and 1.6 g of starch resulted in electrolytes with gel-like nature. Further addition of starch (above 1.6 g) resulted in sedimentation, and a solid character started to appear. So, in this work, we are presenting the data near to the point of gel-type electrolytes (i.e., electrolyte containing 1.4 and 1.6 g starch and liquid electrolyte). The prepared electrolytes are shown in Figure 2.

**Figure 3:**
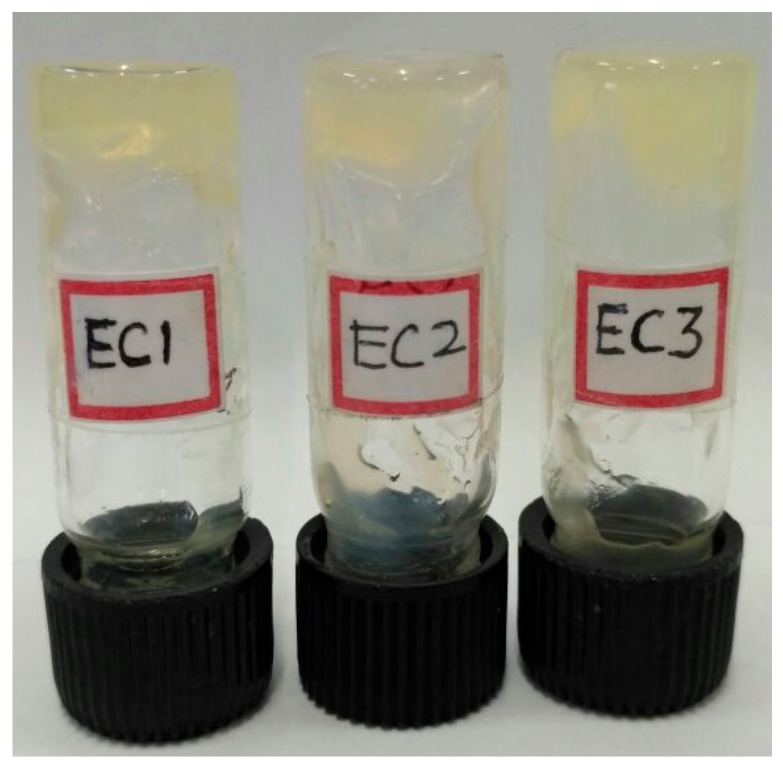
Gel polymer electrolytes (EC1, EC2, EC3 are electrolytes prepared with A1, A2, and A3, respectively) prepared through cross-linked starch.

Further, for the preparation of cross-linked electrolytes, 1.4 g was taken as the optimum weight and the electrolytes were prepared by following the procedure mentioned above. The prepared gel polymer electrolytes are shown in Figure 3.

### 2.6. Cell Fabrication

DSSCs were fabricated by sandwiching the prepared biopolymer gel and liquid electrolytes in between the photoanode (FTO/TiO_2_/N719) and a platinized counter electrode. To maintain the thickness of the electrolyte layer and to avoid short-circuiting between the two conductive glass surfaces, the two electrodes were brought in close proximity with the help of an adhesive tape of thickness ~50 µm.

### 2.7. Characterization Techniques

Fourier transform infrared spectra (FT-IR) of the electrolytes were obtained with a FT-IR spectrometer (Nicolet iS5, ThermoFisher Scientific, Waltham, MA USA.) and X-ray diffraction spectroscopy was done using X-ray diffractometer (Cu-Kα radiation, Rigaku Corporation, Tokyo, Japan). Differential scanning thermograms were obtained with a Differential scanning calorimeter (model DSC6000, PerkinElmer, Waltham, MA USA.) instrument at the heating rate of 5 °C min^−1^. All electrochemical measurements were conducted using an electrochemical analyser (AUTOLAB12/FRA2, Metrohm, Herisau, Switerland). The conductivity of the gel polymer electrolytes was determined by electrochemical impedance spectroscopy (EIS) by sandwiching the electrolyte between two platinum-coated conductive FTO glasses at a distance of about 50 µm, and the conductive side of one plate was properly masked with an insulating tape, leaving an open area of 1 cm^2^. The photovoltaic properties of the fabricated solar cells were determined by illuminating under a solar simulator from a 150 W Xe light source in combination with standard AM1.5 (85 mW cm^−2^). The electrochemical impendence spectra of the cells were obtained by forward bias at open circuit voltage in dark conditions from 1 Hz to 10^6^ MHz with a perturbation voltage of 10 mV.

## 3. Results

**Figure 4:**
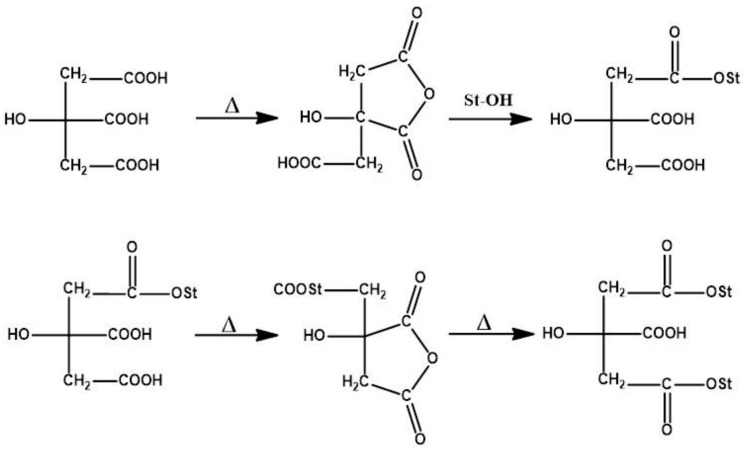
Mechanism of cross-linking of starch with citric acid.

The cross-linking reaction mechanism of starch with citric acid is given in Figure 4 [16,17]. Citric acid will form ester groups with –OH groups of the starch molecule during cross-linking and get inserted into the polymer chain and introduce hindrance to the iodine molecule. Thus, the formation of the starch–iodine complex is prevented. The cross-linking also decreases the water permeability of starch and leads to a more stable electrolyte [13].

### 3.1. Fourier-Transform Infrared Spectroscopy

**Figure 5:**
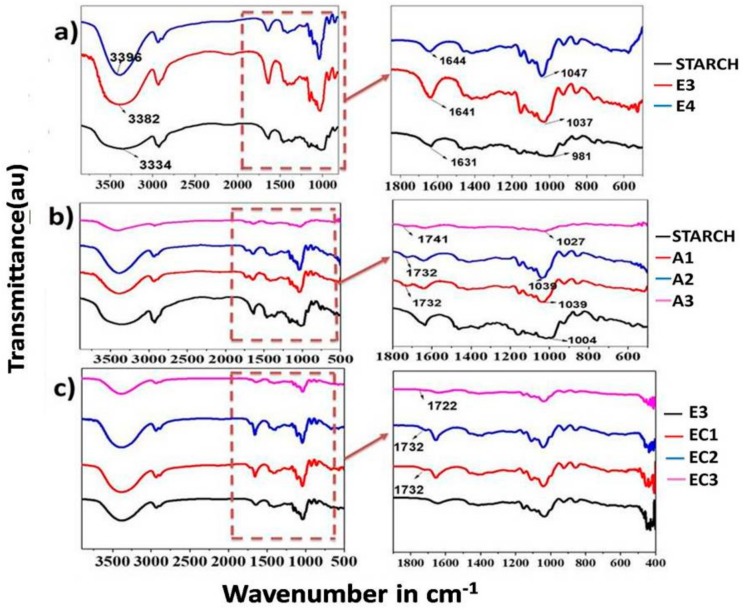
Infrared spectra of (**a**) native starch and gel polymer electrolyte containing 1.4 g (E3) and 1.6 g (E4) of bare starch; (**b**) native starch and cross-linked starches A1, A2, and A3; (**c**) electrolytes E3, EC1, EC2, EC3 prepared using bare starch and cross-linked starch A1, A2, and A3, respectively.

The FTIR analysis provides a powerful means to characterize the complex formation between the components in the polymer electrolyte. FTIR spectra of native starch and the prepared electrolytes are shown in Figure 5. The bands at 925 cm^−1^ are attributed to the skeletal mode of vibrations of 1,4-α-glycosidic linkages (C-O-C). Water adsorbed in the amorphous region of starches could be identified as a broad infra-red band at 1637 cm^−1^; this band becomes weaker if the crystalline nature of starch increases [18]. In the enlarged view of the IR spectra, the peak at 1636 cm^−1^ for bare starch is very weak, and it shows the crystalline nature. However, this peak shifted to 1641 cm^−1^ for E3 and to 1644 cm^−1^ for E4 electrolyte (containing 1.4 g and 1.6 g starch respectively) and became sharper, illustrating the increase in amorphous nature. The introduced amorphous regions in the electrolytes result in the improvement of the ionic conductivity of the electrolytes. The C-H stretching mode of vibrations is visible at 2920–2930 cm^−1^ [19]. The O-H stretching mode of vibration is found at 3379 cm^−1^ in the spectrum of starch, which became more intense and shifted to a higher frequency for E3 (3382 cm^−1^) and E4 (3396 cm^−1^), showing the strengthening of the –OH bond which is attributed to the interaction of starch molecules with glycerol [19].

Figure 5b showed the FTIR spectrum of cross-linked starches A1, A2, and A3. All three samples show similar peaks at 927, 860, and 578 cm^−1^, which are attributed to the stretching vibrations of the whole glucose ring. The peak at 3407 cm^−1^ of the esterified starch, which corresponds to the stretching vibration of –OH group [20], decreased in intensity compared to the native starch, indicating that parts of the –OH group of starch are esterified. In addition, a new peak at 1736 cm^−1^ which corresponds to C=O stretching in the ester group was observed for cross-linked starches, which proves the cross-linking of starch with citric acid in A1, A2, and A3. A peak observed at 1003 cm^−1^ is due to stretching vibration of C-O bond in the anhydrous-glucose ring in bare starch. In the case of cross-linked starches, the above peak shifted to 1034 cm^−1^ and this shows the weakening of hydrogen bond interactions of starch due to the introduction of sterically hindered ester bonds during crosslinking. 

From Figure 5c, FT-IR spectra of gel electrolytes containing cross-linked starch are similar to the results found for cross-linked starch in the literature [20,21]. The ester peaks are visible in the spectra of electrolytes, which shows that the cross-linking was also maintained in the electrolytes. The decrease in the intensity of the peaks of –OH groups shows that the lesser interaction of cross-linked starch with glycerol is mainly due to the lack of available –OH groups of starch for hydrogen bonding, which are esterified during cross-linking reaction.

### 3.2. X-ray Diffraction

**Figure 6:**
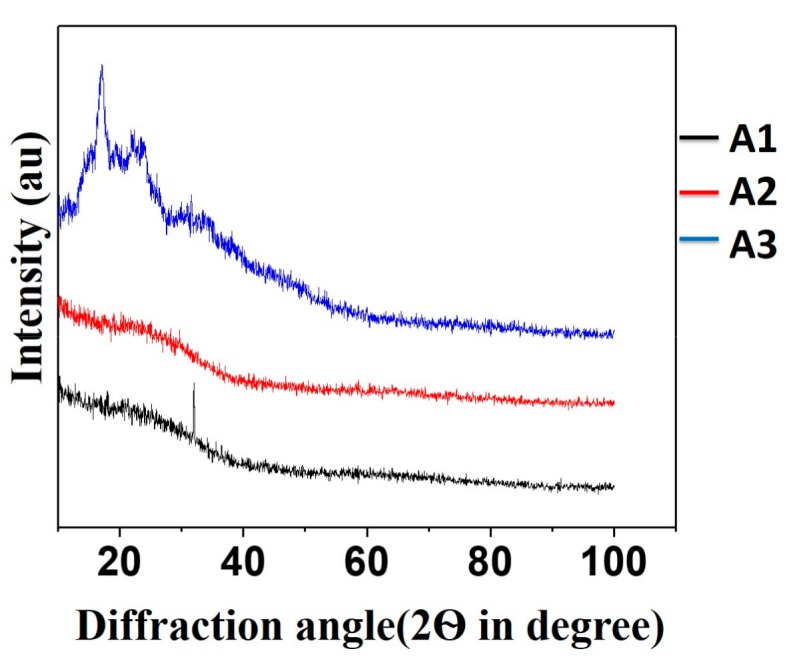
X-ray diffraction pattern of cross-linked starches A1, A2, and A3.

Starch having many –OH groups in its structure will remain in intermolecular hydrogen bonding with the –OH groups of glycerol. When the cross-linking agent citric acid is added, it will form an ester linkage with available –OH groups in starch and help to increase the amorphous nature of starch [22]. XRD graphs of modified starch are shown in Figure 6.

The characteristic diffraction peaks were observed at 17.8°, 21.8°, and 24.1° for starch granules (in Figure S1) [23]. In the XRD graphs of cross-linked starch, it can be seen that the above characteristic peaks disappear in the diffractogram, which illustrates the loss of the crystalline nature of starch as a result of cross-linking.

### 3.3. Differential Scanning Calorimetry (DSC)

**Figure 7:**
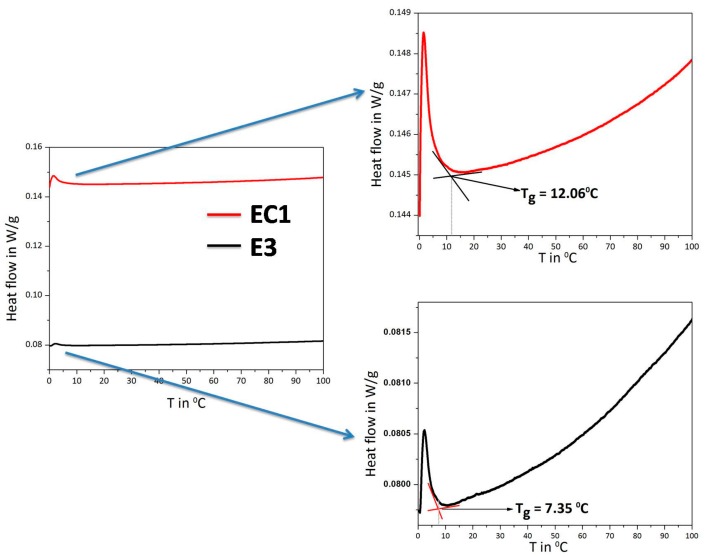
Differential scanning thermogram of an electrolyte containing bare starch (E3) electrolyte (EC1) containing cross-linked starch A1.

The molecular motions of polymer chains are inhibited at low temperatures and increase when thermal energy is applied. The temperature at which the physical change of crystalline to flexible state takes place is called the glass transition temperature (*T*_g_) [24]. The inflection points in DSC thermograms of the electrolytes (Figure 7) are measured as *T*_g_. It is reported that the introduction of cross-links deprives the segmental motion of polymers and increases *T*_g_ due to the reduction in the number of chain units that could be thermally activated. *T*_g_ is an indirect measure of cross-link density [25]. From Figure 7, upon cross-linking of starch, the *T*_g_ of gel electrolytes is shifted to higher temperature when compared with bare starch-based electrolyte due to the restricted segmental mobility of starch by cross-linking with citric acid [26].

### 3.4. Scanning Electron Microscopy

**Figure 8:**
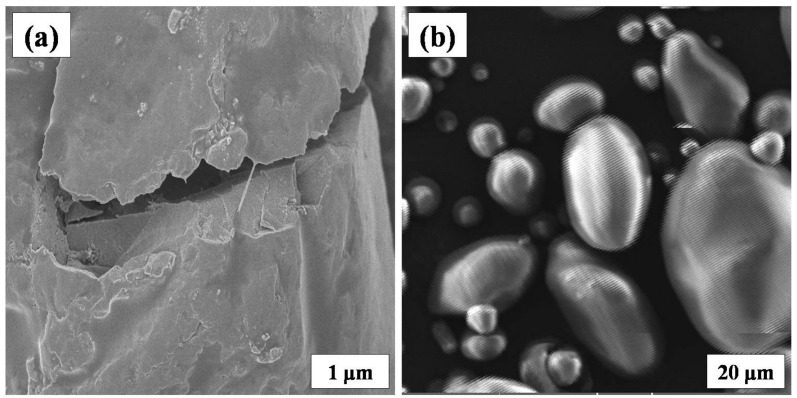
(**a**) SEM image of gel polymer electrolytes (E3) containing 1.4 g bare starch; and (**b**) granular structure of native starch.

From Figure 8, it is clear that in starch-based gel electrolytes (1.4 g) the basic granular structure (Figure 8b) of starch is destroyed. Additionally, the electrolyte layer is more homogeneous because of the interaction of starch with glycerol through hydrogen bonding interactions.

### 3.5. Conductivity Measurements of Prepared Gel Polymer Electrolytes

The efficiency of the DSSC composed of gel polymer electrolyte is highly influenced by the mobility of ions, and hence we measured the conductivity of electrolytes [27]. The conductivities (*σ*) of the prepared gel electrolytes are calculated from the measured bulk resistance (*R*_b_) values obtained in electrochemical impendence spectroscopy (Figure S2 and equivalent circuit in Figure S3) by using the following relation:

(1) $$\sigma =\frac{l}{R_{b}A}$$

where “*σ*” is the ionic conductivity, “*l*” is the distance between two electrodes, “*R*_b_” is the bulk resistance (diameter of the first semicircle), and “*A*” is the active area, which is taken as 1 cm^2^. The equivalent circuit used and the circuit elements value are shown in the supplementary information. From Table 1, the conductivity value of the gel type electrolytes was lower than that of liquid-based electrolyte due to the ease of ionic motion in a liquid medium. Among gel electrolytes containing bare starch, E3 showed the maximum ionic conductivity of 1.61 S cm^−1^. Further, the conductivity decreased on cross-linking compared to E3, possibly due to the lack of –OH groups to interact with the –OH groups of glycerol. Additionally, as mentioned in the Section 3.3, segmental mobility is restricted as a result of cross-linking, and consequently ionic mobility is decreased. Moreover, the ionic conductivities of electrolytes EC2 and EC3 were lower compared to EC1, possibly due to the co-existence of Na^+^ in the electrolyte medium containing Li^+^ [28].

### 3.6. Photovoltaic Measurements

**Figure 9:**
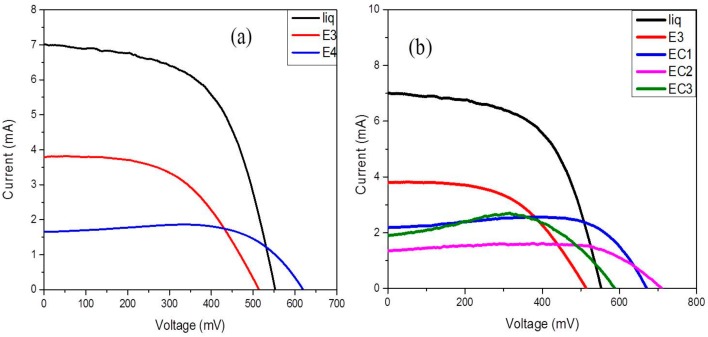
I–V curves of dye-sensitized solar cells (DSSCs) composed of (**a**) liquid electrolyte and gel electrolytes containing 1.4 g (E3) and 1.6 g (E4) of bare starch; (**b**) gel electrolytes E3, EC1, EC2, and EC3 containing 1.4 g bare starch and cross-linked starches A1, A2, and A3 respectively.

The photocurrent-voltage measurements of the fabricated DSSC composed of liquid, bare starch, and cross-linked starch under 85 mW cm^−2^ (1.5 AM solar simulator) are shown in Figure 9. Experimental results are also tabulated in Table 2. The efficiency of a DSSC depends upon three main factors: (i) open circuit voltage (*V*_OC_), (ii) short circuit current (*J*_SC_), and (iii) fill factor (ff) of the cell. *J*_SC_ mainly depends upon ionic mobility in the electrolyte, *V*_OC_ depends upon the recombination resistance and electron lifetime of electrons in the conduction band of TiO_2_ [29], and ff depends on uniformity and pore filling ability of electrolyte layer.

From Table 2, the DSSC containing liquid electrolyte had higher efficiency compared to that of gel electrolyte, and in bare starch-based gel electrolytes, E3 showed better efficiency compared to E4 because of higher short circuit current (*J*_SC_) values due to high ionic conductivity. Apart from the lower value of conductivity (*σ*), EC1 showed better photocurrent conversion efficiency and *V*_OC_ compared to E3. This may be due to the reduction in charge recombination reaction in the presence of cross-linked starch. This is further confirmed by EIS and electron lifetime measurements in the following sections. 

In the case of EC2 and EC3, the presence of sodium ions (Na^+^) introduced in the form of Na_2_CO_3_ and NaOH in EC2 and EC3, respectively, during cross-linking significantly influenced the performance of the DSSC and resulted in lower *J*_SC_ values, probably due to the formation of ionic clusters, leading to the decrease in the effective concentration of free charge carriers [30]. The presence of Na^+^ cation along with Li^+^ in electrolyte decreased the relative quantum yield for electron injection (φ_inj_) from LUMO of dye to the conduction band of TiO_2_. All of these factors may contribute to the lower performance of the DSSCs composed of EC2 and EC3 compared to the DSSC composed of EC1.

### 3.7. Electrochemical Impedance Spectroscopy

**Figure 10:**
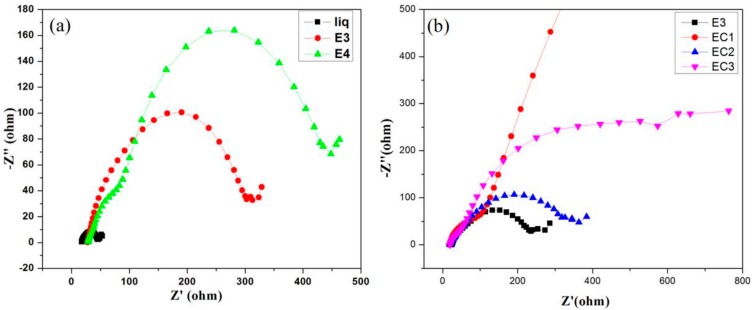
Nyquist plots from electrochemical impedance spectroscopy (EIS) showing the electron recombination resistance of DSSCs composed of (**a**) liquid electrolyte and gel electrolytes containing 1.4 g (E3) and 1.6 g (E4) of bare starch; (**b**) gel electrolytes E3, EC1, EC2, and EC3 containing 1.4 g bare starch and cross-linked starches A1, A2, and A3 respectively.

The interfacial resistance and electronic transport process of the DSSC were studied by the electrochemical impedance spectroscopic (EIS) analysis. Figure 10 shows the Nyquist plots from EIS measurement of DSSCs and includes three semi-circles [31]. The first semi-circle in the low-frequency range represents the Pt/electrolyte interface, and it is independent of light intensity. The second light-dependent semi-circle at the mid-frequency (between 1 to 100 Hz) is associated with the TiO_2_/dye/electrolyte interface. The increment in the diameter of this semi-circle implies the higher charge transfer resistance (*R*_CT_) at the TiO_2_/dye/electrolyte interface. The last semi-circle at the high-frequency region corresponds to Warburg resistance for tri-iodide diffusion, *Z*_W_. From Table 2, it is clear that that the *R*_CT_ increases with the increase in the concentration of starch. Further, *R*_CT_ is higher for DSSC composed of EC1 and accounts for higher open-circuit voltage and efficiency of DSSC.

### 3.8. Electron Lifetime Measurements

**Figure 11:**
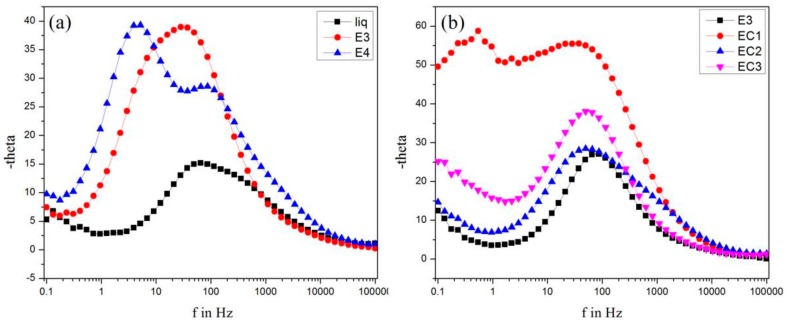
Bode phase plots of DSSCs composed of (**a**) liquid electrolyte and gel electrolytes containing 1.4 g (E3) and 1.6 g (E4) of bare starch; (**b**) gel electrolytes E3, EC1, EC2, and EC3 containing 1.4 g bare starch and cross-linked starches A1, A2, and A3 respectively.

The Bode phase plots of DSSC composed of different electrolytes are shown in Figure 11. Bode phase plots give the frequency responses of a system with phase change. From the plots, the peak of each graph is taken as the maximum frequency (*f*_max_) and used for the calculation of electron lifetime at the conduction band of TiO_2_ using Equation (2):

(2) $$\tau =\frac{1}{2\pi f}$$

where “*τ*” is the electron lifetime and “*f*” is the maximum frequency. The electron lifetime values are given in Table 2. Electron lifetime measurements are in good agreement with recombination resistance values; i.e., *τ* increases with the increase in the concentration of starch and is higher for DSSC composed of EC1, showing the reduction in back electron transfer by the addition of starch and by cross-linking [32].

## 4. Conclusions

A new starch-based biopolymer gel electrolyte is prepared for the application of a dye-sensitized solar cell. The effects of preparation methods, as well as the degree of cross-linking on the physical properties and photoconversion efficiency of the resulting DSSC cells, are carefully analyzed. FT-IR spectroscopy of the gel polymer electrolytes shows the conformational changes of starch during cross-linking. X-ray diffraction reveals that the cross-linking processes affect the quality and quantity of the crystalline nature of starch. Cross-linking in starch also changes its glass transition temperature, as proved by differential scanning calorimetry. DSSCs composed of prepared electrolytes are characterized by electrochemical and photovoltaic measurements. The newly prepared electrolyte shows an ionic conductivity of 1.61 S cm^−1^ and DSSC with 1.2% efficiency. Cross-linking improved the electron lifetime and recombination resistance of DSSC and the photoconversion efficiency to 1.4% with conductivity 0.59 S cm^−1^. Results clearly show additional potential for further cost reduction and simplification of the manufacturing of dye solar cells.

## Figures and Tables

**Table 1:** Table showing the bulk resistance and ionic conductivity of the prepared electrolytes.

Electrolyte	*R*_b_ (ohm)	Conductivity (S cm^−1^)
Liquid	29.74	1.72
E3	30.01	1.61
E4	31.53	1.58
EC1	84.69	0.59
EC2	129.15	0.38
EC3	164.1	0.30

**Table 2:** Table showing the open circuit voltage, short circuit current, fill factor, efficiency, recombination resistance, and lifetime measurements of liquid, E3, E4, EC1, EC2, and EC3 electrolytes.

Electrolyte	*V*_OC_	*J*_SC_	FF (%)	*η* (%)	*R*_CT_ (ohm)	*τ* (s)
Liquid	552.41	7.06	57	2.6	33.82	2.2 × 10^−3^
E3	514.77	3.98	50	1.2	184.12	3.2 × 10^−3^
E4	624.72	1.55	78	0.89	277.97	3.5 × 10^−3^
EC1	672.02	2.17	82	1.4	308.62	3.72 × 10^−3^
EC2	708.46	1.33	84	0.93	193.80	3.52 × 10^−3^
EC3	586.53	1.89	85	1.1	302.95	3.48 × 10^−3^

## Supplementary Materials

The following are available online at www.mdpi.com/2073-4360/9/12/667/s1, Figure S1: X-ray diffraction pattern of native starch; Figure S2: Electrochemical impedance graphs of (a) electrolytes containing 1.4 g (E3) and 1.6 g (E4) starch and liquid electrolyte (b) electrolytes E3, EC1, EC2 and EC3 containing 1.4 g bare starch, A1, A2 and A3 respectively; Figure S3: Equivalent circuit used to extract electrochemical impedance data, Table S1: Values of the circuit elements obtained using equivalent circuit.
