# Supplementary material for: Effect of Cross-Linking on the Performances of Starch-Based Biopolymer as Gel Electrolyte for Dye-Sensitized Solar Cell Applications
Source: Polymers (Basel). 2017 Dec 1;9(12):667. doi: 10.3390/polym9120667 (PMC6418899; doi:10.3390/polym9120667)
Supplement: Supplementary file 1 [file polymers-09-00667-s001.pdf]

Effect of Cross-linking on the Performances of Starch-Based Biopolymer as Gel Electrolyte for Dye-Sensitized Solar Cell Applications

Pavithra Nagaraj [1], Asija Sasidharan [1], Velayutham David [2] and Anandan Sambandam [1,*]

[1] Nanomaterials and Solar Energy Conversion Lab, Department of Chemistry, National Institute of Technology, Tiruchirappalli 620 015, India; pavithra1516@gmail.com (P.N.); asijasasidharan@gmail.com (A.S.)

[2] Electro Organic Division, CSIR-Central Electrochemical Research Institute, Karaikudi 630 006, India; dvelayutham@cecri.res.in

* Correspondence: sanand@nitt.edu.; Tel.: +91-431-250-3639

Supplementary information

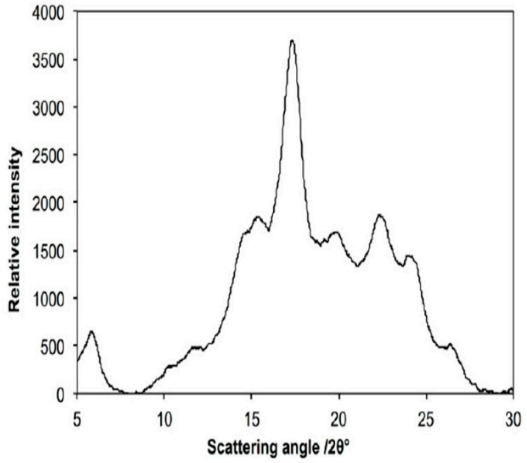

Figure S1. X-ray diffraction pattern of native starch [1].

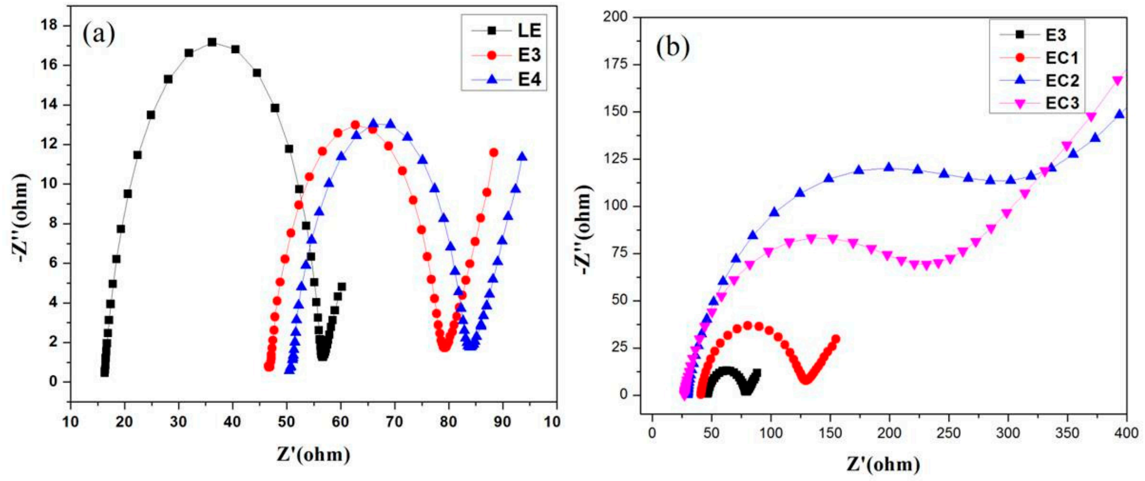

Figure S2. Electrochemical impedance graphs of (a) electrolytes containing 1.4g (E3) and 1.6g (E4) starch and liquid electrolyte (b) electrolytes E3, EC1, EC2 and EC3 containing 1.4g bare starch, A1, A2 and A3 respectively.

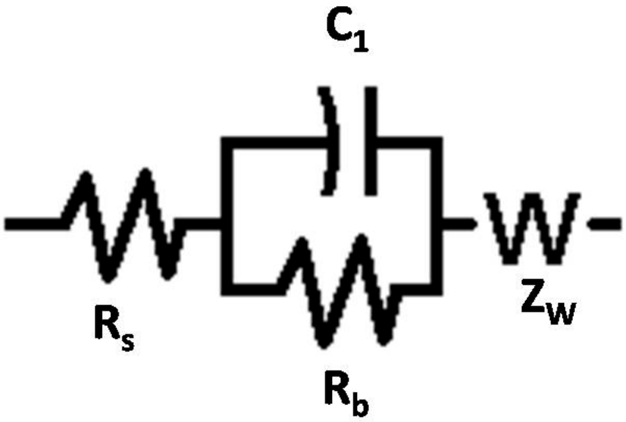

Figure S3. Equivalent circuit used to extract electrochemical impedance data.

Table S1. Values of the circuit elements obtained using equivalent circuit.

Electrolyte	R_S (ohm)	R_b (ohm)	C_1×E-6 (nF)	Z_W (ohm)
Liquid	16.94	29.74	8.13	0.1632
E3	47.70	30.01	8.21	0.0781
E4	51.78	31.53	8.83	0.0759
EC1	41.87	84.69	9.83	0.0267
EC2	28.70	129.15	15.20	0.0015
EC3	27.24	164.1	14.09	0.0026

References

1. Das, K; Ray, D; Bandyopadhya, N.R; Gupta, A; Sengupta, S; Sahoo, S; Mohanty A; Misra M. Preparation and Characterization of Cross-Linked Starch/Poly(vinyl alcohol) Green Films with Low Moisture Absorption. *Ind. Eng. Chem. Res.*, **2010**, 49, 2176–2185.doi: 10.1021/ie901092n.
